# Ultrasound Evaluation of Knee Osteoarthritis

**DOI:** 10.7759/cureus.39188

**Published:** 2023-05-18

**Authors:** Rock P Vomer, Samuel Boggess, Blake Boggess

**Affiliations:** 1 Department of Family and Community Health/Department of Orthopedics, Division of Sports Medicine, Duke University, Durham, USA; 2 Family Medicine, Mayo Clinic Jacksonville Campus, Jacksonville, USA; 3 Department of Osteopathic Medicine, Philadelphia College of Osteopathic Medicine-Georgia, Suwanee, USA; 4 Department of Orthopedics, Division of Sports Medicine, Duke University, Durham, USA

**Keywords:** ultrasound protocol, clinical monitoring, cost reduction, ultrasound, osteoarthritis, knee osteoarthritis

## Abstract

While radiographs and magnetic resonance imaging (MRI) have long been used in the assessment of osteoarthritis (OA), ultrasound imaging has been rapidly accepted by musculoskeletal providers in both the assessment and treatment of OA. A limiting factor in the use of ultrasound is the proper training required by the user for results to be reliable and reproducible. A standardized ultrasound protocol can potentially address this limiting factor. The critical information to consider in a standardized protocol include proper patient positioning, probe alignment, probe orientation, and identification of the appropriate anatomic landmarks. The outlined protocol considers these factors with the purpose of providing a step-by-step method to assess and monitor knee OA.

## Introduction

Osteoarthritis (OA) is the most prevalent degenerative joint disease, reflecting considerable ramifications in the assessment and treatment of chronic pain and disability [1]. Knee OA can affect the medial, lateral, and patellofemoral joint and usually develops slowly over time, and results in decreased function with activities of daily living. The degeneration that occurs with knee OA is multifactorial with both inflammatory and biomechanical processes cooccurring. It is also influenced by a combination of factors, that include family history, age, obesity, lower limb alignment, joint shape, trauma, and chronic inflammation [2]. The risk factors related to the development of knee OA can be divided into two categories: nonmodifiable and modifiable. Nonmodifiable risk factors include hereditary and congenital abnormalities that affect the bone, cartilage, and connective tissue of the knee. Modifiable risk factors are able to be adjusted and the most common is excess body weight [2]. Excess weight increases joint loading, and for every pound of weight gained the knee is exposed to an extra two to four pounds of extra force [2,3]. Thirteen percent of women and 10% of men aged 60 years and older have symptomatic knee OA [4]. The proportion of people affected with symptomatic knee OA is likely to increase due to the aging of the population and the rate of obesity or overweight in the general population [5].

Although OA can be diagnosed clinically, diagnostic imaging of standard radiographs and magnetic resonance imaging (MRI) are traditionally used to identify structures involved and monitor progression [2,3]. The disadvantage of this traditional approach is the combination of cost, time involved, access to the modalities, and the static nature of the images. Ultrasound is an imaging modality that can be used to address these disadvantages. Ultrasound is inexpensive, noninvasive, readily available, and reliably produces dynamic images of the inflammatory changes in knee OA [2,4,6,7].

Ultrasound is a user-dependent tool and requires proper training for its results to be reliable and reproducible [8]. As ultrasound use continues to grow in acceptance and incorporation into medical training, standardized protocols can improve users' skills [9]. Developing a standardized protocol can also enhance diagnostic accuracy and improve communication among peers in the medical community. While prior publications have reported the benefit of standardized scan protocols, step-by-step protocols for assessing knee OA have not been outlined [10-12]. The purpose of the report is to provide a step-by-step method for patient positioning, probe alignment, probe orientation, and landmark identification in the assessment and monitoring of knee OA. The protocol is divided into anterior, medial, lateral, posterior, and standing evaluations of the knee.

## Technical report

Anterior evaluation

**Figure 1:**
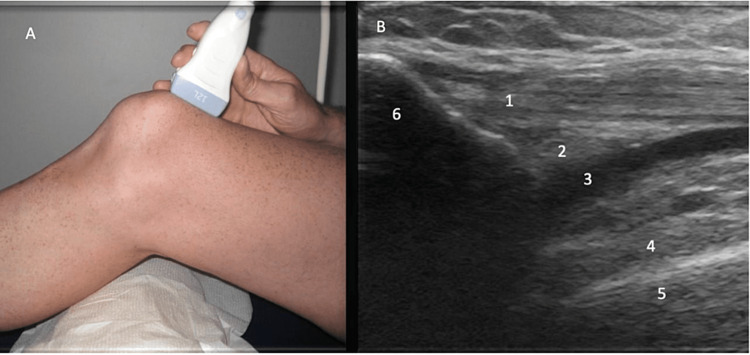
Anterior Evaluation, Long Axis View Figure 1A: The patient is placed in a supine with a bolster under the posterior knee to flex the knee to 30˚. The probe is aligned parallel to the quadriceps tendon. Figure 1B: The quadriceps tendon (1), quadriceps fat pad (2), suprapatellar recess (3), prefemoral fat pad (4), femur (5) and patella (6) are identified.

**Figure 2:**
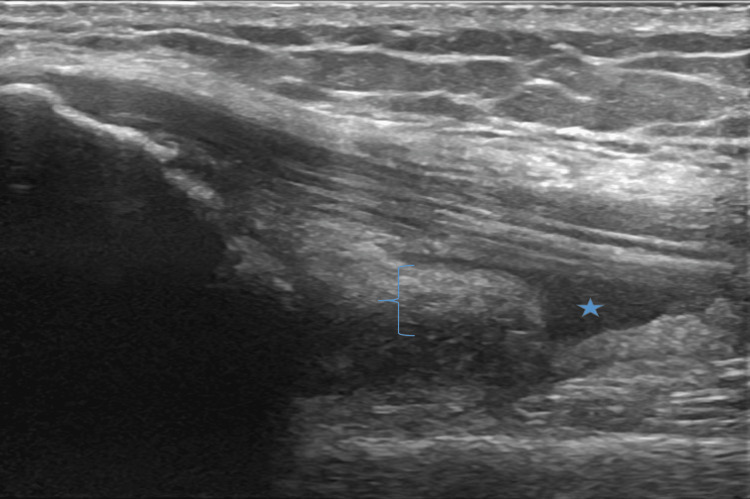
Long Axis View of Anterior Knee in the Setting of Osteoarthritis. Ultrasound image of a knee with osteoarthritis demonstrating hypoechoic effusion (star) and synovitis (bracket).

**Figure 3:**
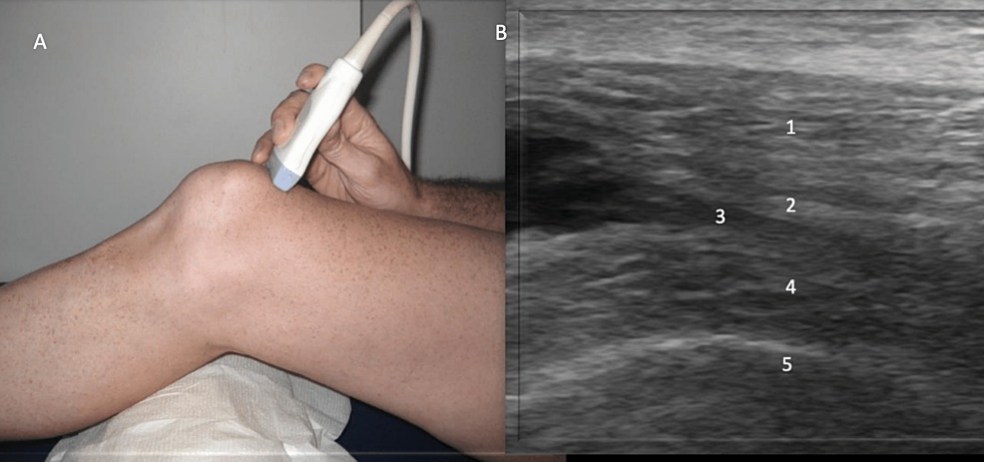
Anterior Evaluation, Short Axis View Figure 3A: The probe is rotated 90˚ perpendicular to the quadriceps tendon. Figure 3B: The quadriceps tendon (1), quadriceps fat pad (2), suprapatellar recess (3), prefemoral fat pad (4), and femur (5) are identified.

**Figure 4:**
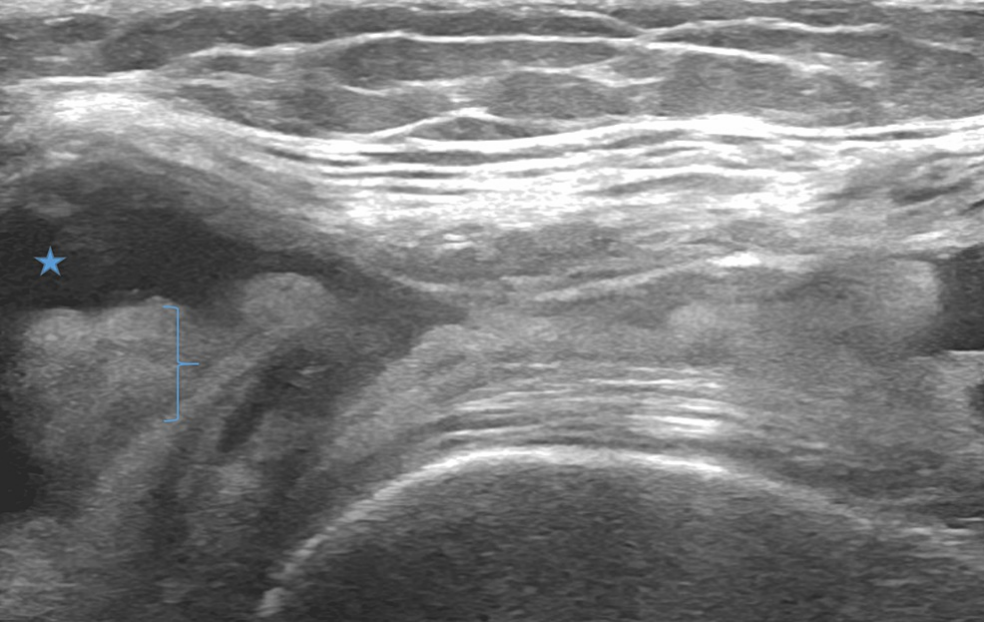
Short Axis View of Anterior Knee in the Setting of Osteoarthritis. Ultrasound image findings that support the diagnosis of osteoarthritis are synovitis (bracket) and hypoechoic effusion (star).

For anterior evaluation, place the patient supine with a bolster under the posterior knee to flex the knee to approximately 30˚. Align the probe parallel to the quadriceps tendon with the distal end of the probe over the base of the patella. Next, identify the quadriceps tendon, quadriceps fat pad, suprapatellar recess, prefemoral fat pad, femur, and patella (Figure 1). In this position observe for joint effusion and synovitis, both of which are signs of OA of the knee (Figure 2). Then rotate the probe from the longitudinal alignment to 90˚ perpendicular to the quadriceps tendon. Identify the quadriceps tendon, quadriceps fat pad, suprapatellar recess, prefemoral fat pad, and femur (Figure 3). When the knee is viewed with ultrasound in the short axis potential signs of OA the ultrasound image that may be observed are synovitis and effusion (Figure 4).

**Figure 5:**
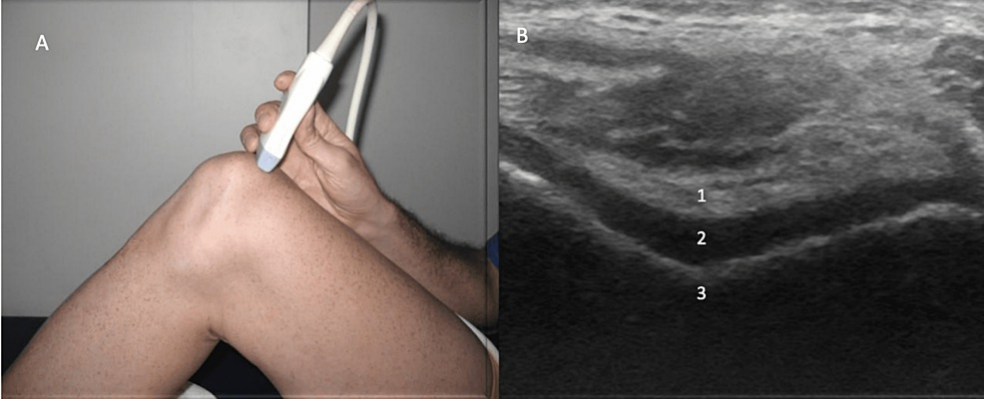
Anterior Evaluation, Maximal Knee Flexion Figure 5A: The knee is placed in maximal flexion with the probe aligned 90˚ perpendicular to the quadriceps tendon. Figure 5B: The quadriceps tendon (1), hyaline cartilage (2) and femur (3) are identified [12].

**Figure 6:**
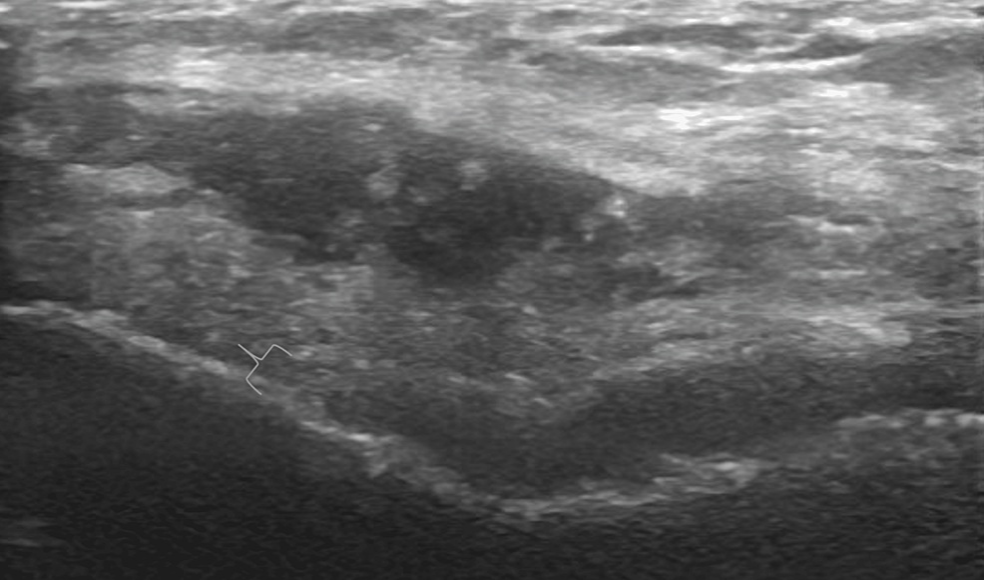
Anterior Evaluation, Maximal Knee Flexion in the Setting of Osteoarthritis. Ultrasound image demonstrating loss of hyaline cartilage (bracket).

**Figure 7:**
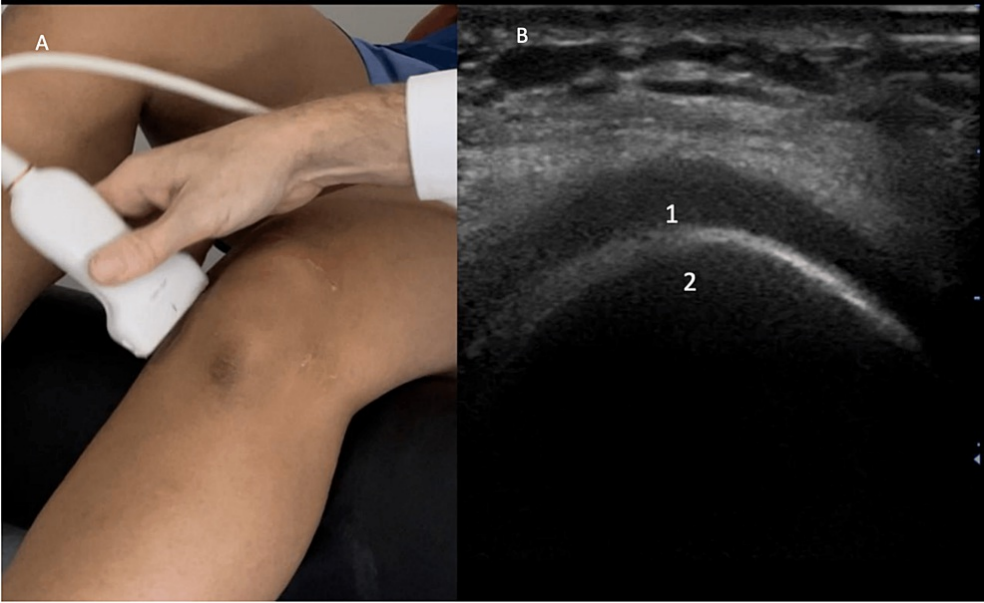
Anterior Evaluation of Medial Cartilage Figure 7A: The probe is positioned medially in longitudinal access over the medial femoral condyle. Figure 7B: The hyaline cartilage (1) and femur (2) are identified.

**Figure 8:**
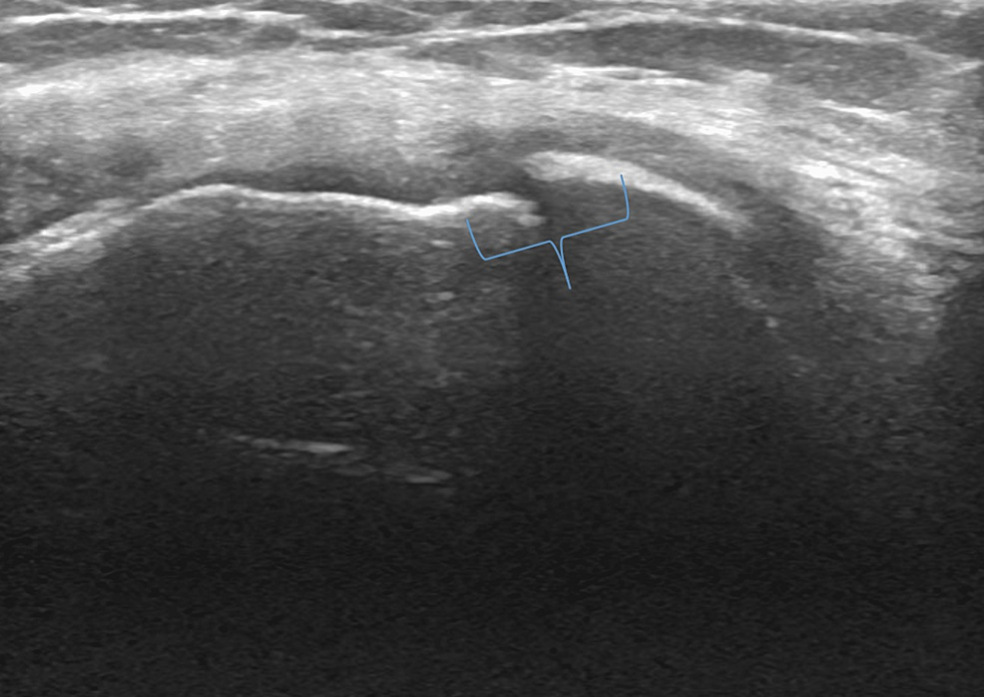
Long Axis View of Medial Condyle in the Setting of Osteoarthritis. Ultrasound image which demonstrates loss of cartilage thickness and cortical irregularities (bracket).

**Figure 9:**
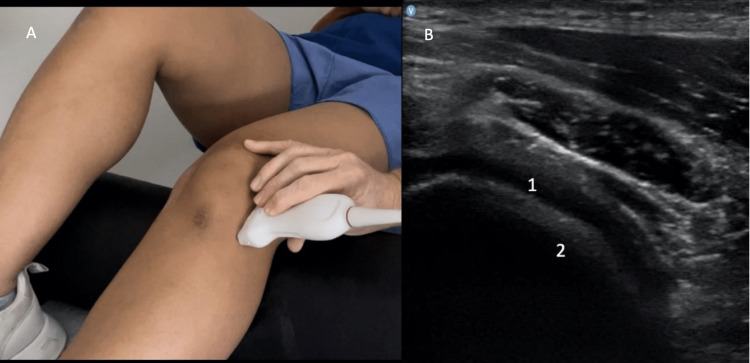
Anterior Evaluation of Lateral Cartilage Figure 9A: The probe is positioned laterally in longitudinal access over the lateral femoral condyle. Figure 9B: The hyaline cartilage (1) and femur (2) are identified.

Once completed, maximally flex the patient's knee and place the probe in a transverse position over the proximal insertion of the quadriceps tendon. Identify the quadriceps tendon, hyaline cartilage, and femur (Figure 5). Once identified, measure the cartilage thickness and identify the presence of calcium deposits. Ultrasound findings supportive of OA are thinning of the hyaline cartilage (Figure 6). Other findings that are consistent with knee OA are cortical irregularities and calcium deposition. Then position the probe medially in longitudinal access over the medial femoral condyle and appreciate the cartilage thickness. Identify the hyaline cartilage and femur (Figure 7). When observing the medial condyle loss in thickness of the articular cartilage and cortical irregularities are supportive of the diagnosis of OA (Figure 8). Osteophytes may also be present in the setting of OA. Then position the probe laterally in longitudinal access over the lateral femoral condyle and appreciate the cartilage thickness (Figure 9).

Medial evaluation

**Figure 10:**
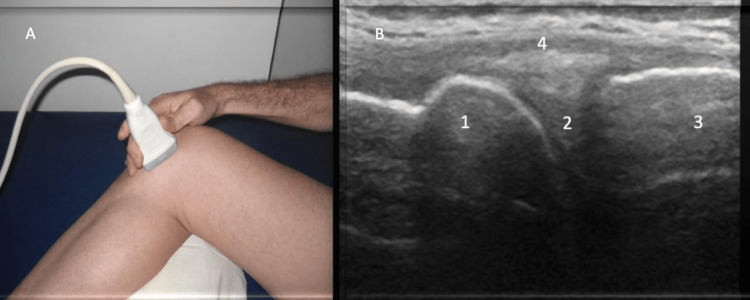
Medial Evaluation, Long Axis View Figure 10A: The knee is repositioned in 30˚ of flexion and the probe is positioned over the medial joint line with the distal aspect of the probe over the proximal tibia. Figure 10B: The distal femur (1), medial meniscus (2), proximal tibia (3), and medial collateral ligament (4) are identified.

For medial evaluation, reposition the knee in 30˚ of flexion. Position the probe over the medial joint line with the distal aspect of the probe over the proximal tibia. Identify the distal femur, medial meniscus, proximal tibia, and medial collateral ligament (Figure 10).

Lateral evaluation

**Figure 11:**
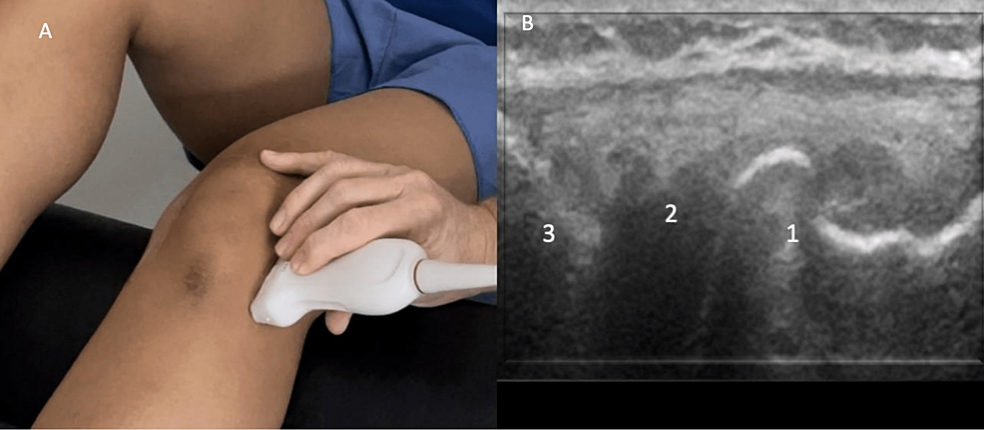
Lateral Evaluation, Long Axis View Figure 11A: The knee remains in 30˚ of flexion and the probe is positioned over the lateral joint line with the distal aspect of the probe over the proximal tibia. Figure 11B: The distal femur (1), lateral meniscus (2), and proximal tibia (3) are identified.

**Figure 12:**
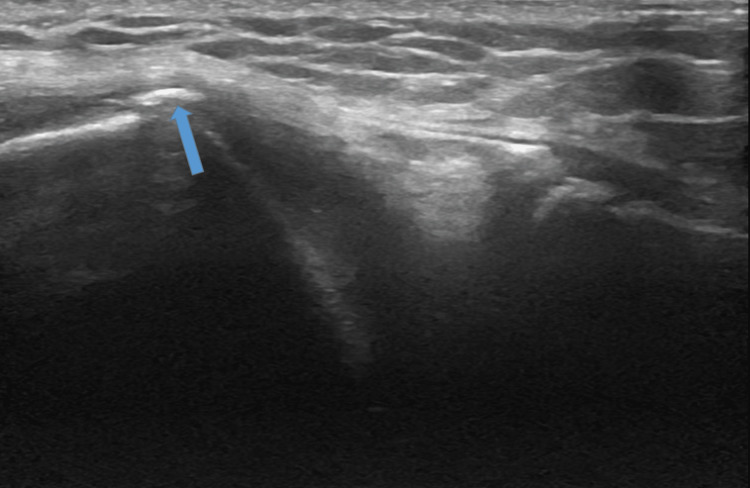
Lateral Evaluation, Long Axis View in the Setting of Osteoarthritis. Ultrasound image demonstrating an osteophyte (arrow) on the lateral condyle.

For lateral evaluation, keep the knee in 30˚ of flexion. Position the probe over the lateral joint line with the distal aspect of the probe over the proximal tibia. Identify the distal femur, lateral meniscus, and proximal tibia (Figure 11). In the setting of knee OA osteophytes and cortical irregularities may be observed on ultrasound examination of the lateral knee (Figure 12).

Posterior evaluation

**Figure 13:**
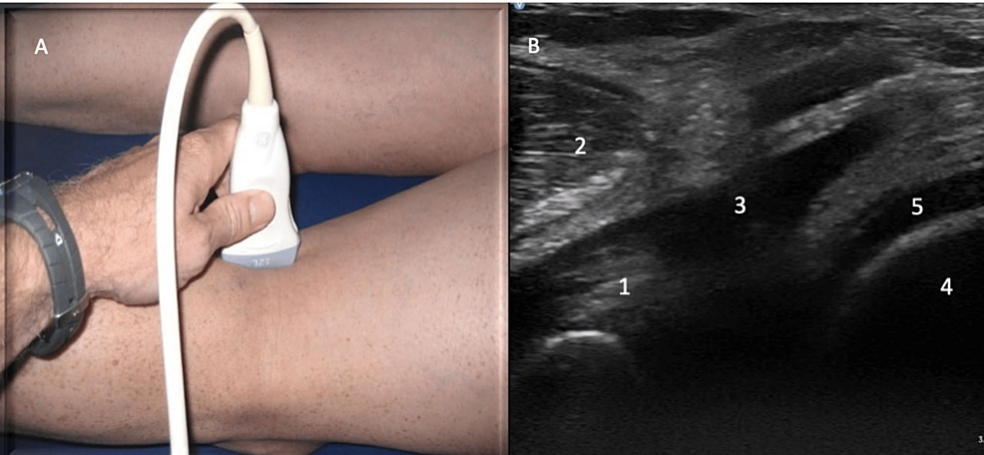
Posteriormedial Evaluation, Long Axis View Figure 13A: The probe is placed over the medial aspect of the popliteal fossa in a longitudinal axis. Figure 13B: The semimembranosus (1), medial head of gastrocnemius (2), presence of a baker’s cyst (3), medial femoral condyle (4), and hyaline cartilage (5) are identified.

For posterior evaluation, position the patient prone with both knees extended. Place the probe over the medial aspect of the popliteal fossa in a longitudinal axis. Identify the semimembranosus, medial head of gastrocnemius, and medial femoral condyle (Figure 13).

**Figure 14:**
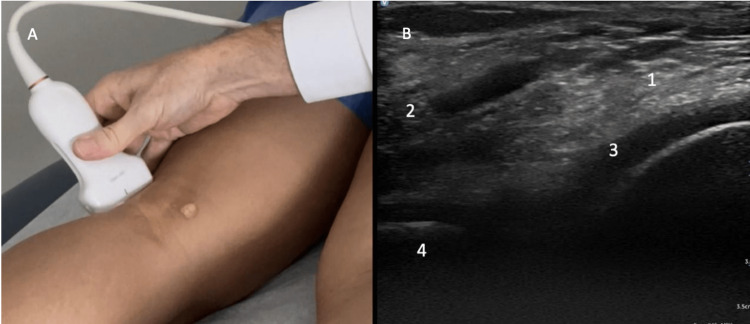
Posteriorlateral Evaluation, Long Axis View Figure 14A: The probe is placed over the lateral aspect of the popliteal fossa in a longitudinal axis. Figure 14B: The semitendinosus (1), lateral head of gastrocnemius (2), lateral femoral condyle hyaline cartilage (3), and lateral proximal tibia (4) are identified.

**Figure 15:**
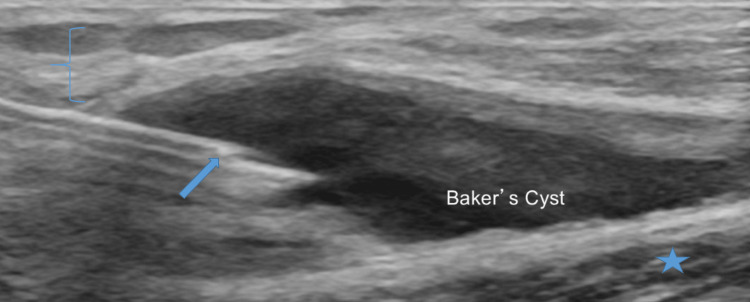
Posteromedial Evaluation, Long Axis View in the Setting of Knee Osteoarthritis. Ultrasound image which demonstrates a needle (arrow) aspirating a hypoechoic fluid collection (Baker’s Cyst) between the semimembranosus (star) and medial head of the gastrocnemius (bracket).

Once completed, rotate the probe 90˚ over the medial femoral condyle and appreciate the thickness of the cartilage. Next, place the probe over the lateral aspect of the popliteal fossa in a longitudinal axis. Identify the semitendinosus, lateral head of gastrocnemius, lateral femoral condyle hyaline cartilage, and lateral proximal tibia (Figure 14). Next, appreciate the thickness of the cartilage of the lateral femoral condyle in a long-axis view. Then rotate the probe 90˚ over the lateral femoral condyle to appreciate the short axis view of the thickness of the cartilage. A common ultrasound finding of the posterior knee which suggests OA is the presence of a hypoechoic fluid collection between the medial head of the gastrocnemius and semimembranosus (Figure 15).

Standing evaluation

**Figure 16:**
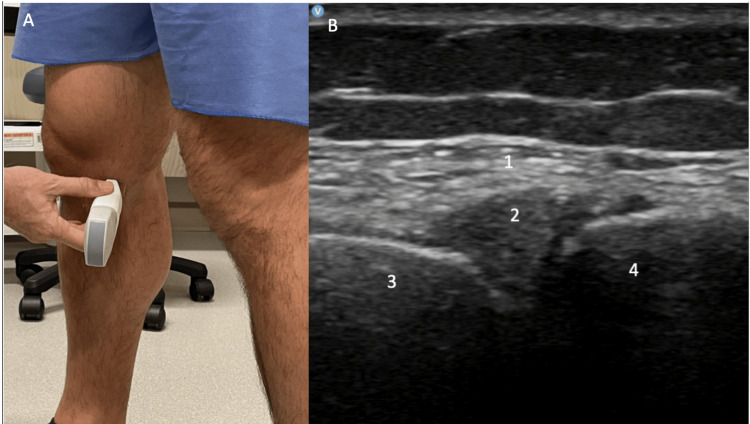
Standing Medial Knee, Extended Long Axis View Figure 16A: The probe is placed over the medial knee joint while the patient is standing, and the knee is extended. Figure 16B: MCL (1), medial meniscus (2), medial femoral condyle (3), and proximal tibia (4) are identified. MCL: medial collateral ligament

**Figure 17:**
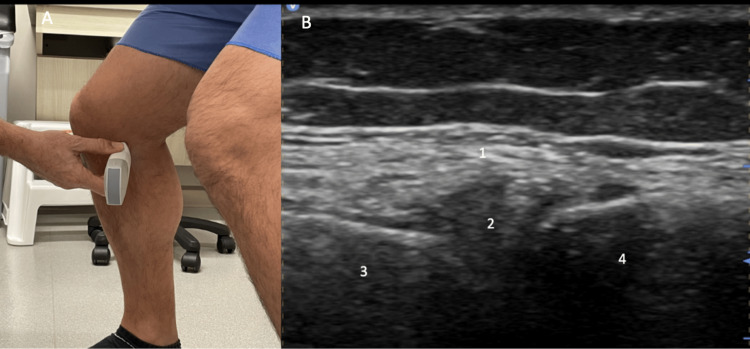
Standing Medial Knee, Flexed Long Axis View Figure 17A: The probe is placed over the medial knee joint while the patient is standing, and the knee is flexed. Figure 17B: MCL (1), medial meniscus (2), medial femoral condyle (3), and proximal tibia (4) are identified. MCL: medial collateral ligament

For standing evaluation, have the patient stand with the knee extended. Place the probe over the medial joint line. Appreciate the medial collateral ligament (MCL), medial meniscus, medial femoral condyle, and proximal tibia (Figure 16). Then place the probe over the medial knee joint with the patient standing and the knee flexed. Compare the difference in meniscal extrusion between the standing and knee flexed position, meniscal extrusion is a sign of OA. Appreciate the MCL, medial meniscus, medial femoral condyle, and proximal tibia (Figure 17).

**Figure 18:**
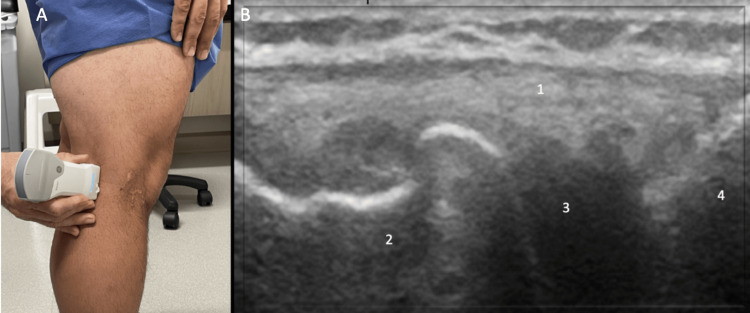
Standing Lateral knee, Extended Long Axis View Figure 18A: The probe is placed over the lateral knee joint while the patient is standing with the knee extended. Figure 18B: Biceps femoris (1), lateral femoral condyle (2), lateral meniscus (3), and proximal tibia (4) are identified.

**Figure 19:**
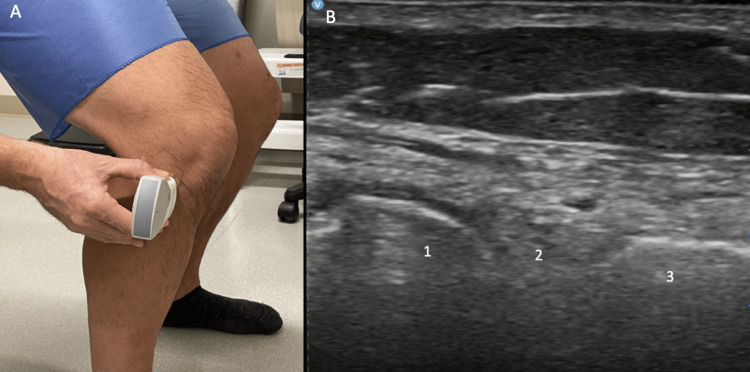
Standing Lateral Knee, Flexed Long Axis View Figure 19A: The probe is placed over the lateral knee joint while the patient is standing with the knee flexed. Figure 19B: Lateral femoral condyle (1), lateral meniscus (2), and proximal tibia (3) are identified.

With the patient still standing and the knee still extended, place the probe over the lateral knee joint. Identify the biceps femoris, lateral femoral condyle, lateral meniscus, and proximal tibia (Figure 18). With the patient still standing, have the patient flex the knee. Place the probe over the lateral knee joint and identify the lateral femoral condyle, lateral meniscus, and proximal tibia (Figure 19).

## Discussion

The critical steps in the ultrasound evaluation of knee OA protocol consist of the proper patient positioning, probe alignment, probe orientation, and identification of the appropriate anatomic landmarks. In each position, the patient should be comfortable and as relaxed as possible. This will allow for the least amount of patient movement during the scan. Proper probe alignment and orientation will help produce clear images to assess bony and soft tissue changes in the knee. Additionally, it will help the provider to reduce anisotropy which could affect image quality. Modifications to the above protocol could be made for patients who are unable to comfortably assume the supine and prone positions outlined above. If a patient is unable to lay flat with just his/her knees bent, additional support could be provided to the lumbar spine and extremities to provide comfort. If a patient is unable to assume a prone position the posterior popliteal evaluations can be obtained from a lateral decubitus position.

The limitations to the ultrasound protocol are user experience and patient motion [13]. To reduce the effect of user experience on image capture quality and accurate interpretation the user should be trained in a standard protocol prior to attempting to utilize ultrasound to monitor knee OA. To reduce the effect of patient motion on capturing quality images, the patient should be comfortably positioned while also instructed to remain still and relaxed during the examination.

To monitor the progression of knee OA, obtain and review ultrasound images of the knee as seen in the figures in the protocol. The review of the images includes the measurement of hyaline cartilage thickness, the presence of osteophyte or calcium deposition, the amount of fluid collection in the joint, and the degree of meniscal extrusion [14]. An effusion is defined as the presence of increased articular fluid at least 4 mm, it will be visualized as a hypoechogenic or an echogenic displaceable material in the knee joint cavity [15]. Overpressure can be used to determine if the hypoechogenic fluid collection is displaceable.

A Baker's cyst is a hypoechogenic fluid collection with clear boundaries of cystic formation situated between the medial gastrocnemius and semimembranosus muscles [16]. It should be assessed in long and short axis and Doppler flow should also be utilized to ensure the structure is not mistaken for a dilated arterial or venous structure [17]. The cyst should also be evaluated for the presence of septations. Ultrasound imaging is able to identify osteophytes, synovitis, and meniscal protrusion reliably in patients who have knee OA. The assessment of articular cartilage is limited to the femoral, trochlear, and condylar cartilage. The presence of these factors is supportive of OA of the knee. Every time new images are obtained, compare them to prior images to assess the progression of OA.

## Conclusions

Utilizing ultrasound to assess knee OA has multiple advantages over traditionally used image modalities but requires user training. The outlined protocol provides a step-by-step method for critical aspects of the use of ultrasound in assessing knee OA. Those aspects are proper patient positioning, probe alignment, probe orientation, and identification of the appropriate anatomic landmarks. The exponentially increased use of ultrasound by musculoskeletal providers gives numerous opportunities for this protocol to be implemented. Future applications for the protocol are to conduct studies investigating the diagnostic accuracy and financial effectiveness associated with ultrasound evaluations for knee OA.
